# Towards fully automated synthetic ECV quantification: an open-access machine learning-based approach for fast blood draw-free CMR

**DOI:** 10.1038/s41598-026-43624-3

**Published:** 2026-03-10

**Authors:** Rebecca Elisabeth Beyer, Markus Hüllebrand, Patrick Doeblin, Ann Laube, Maximilian Leo Müller, Christian Stehning, Stefanie Maria Werhahn, Wensu Chen, Anja Hennemuth, Sebastian Kelle

**Affiliations:** 1 Department of Cardiology, Angiology and Intensive Care Medicine, Deutsches Herzzentrum der Charité, Augustenburger Platz 1, 13353 Berlin, Germany; 2 Charité - Universitätsmedizin Berlin, Corporate Member of Freie Universität Berlin and Humboldt-Universität zu Berlin, Berlin, Germany; 3 DZHK (German Centre for Cardiovascular Research), Partner Site Berlin, Berlin, Germany; 4 Institute of Cardiovascular Computer-Assisted Medicine, Charité – Universitätsmedizin Berlin, Berlin, Germany; 5 Cardiovascular Research and Development, Fraunhofer MEVIS, Bremen, Germany; 6 Philips Clinical Science, Hamburg, Germany; 7 Department of Cardiology, Affiliated Hospital of Xuzhou Medical University, Xuzhou, China

**Keywords:** Cardiology, Computational biology and bioinformatics, Diseases, Health care, Medical research

## Abstract

Extracellular volume (ECV) quantification involves time-consuming multi-step post-processing and a blood draw for hematocrit analysis. This study aimed to develop a fully automated blood draw-free, machine learning-based approach for synthetic ECV assessment for non-invasive assessment of diffuse myocardial fibrosis. We retrospectively evaluated a large clinical cohort of 1092 patients who underwent CMR and ECV measurement at 1.5T or 3T. Participants were divided into training (n = 767) and validation (n = 325) cohorts. Manual contouring of T1 maps was used to iteratively develop a neural network segmentation model, which was then applied for automated analysis. Fully-automated synthetic ECV was calculated using validated sex- and field strength-specific models. Agreement was assessed using Student’s t-test, Pearson correlation, Bland–Altman analysis, and classification analysis. Fully-automated synthetic ECV showed strong correlation with conventional ECV (r = 0.79, *p* < 0.001), with no significant differences (26.9% ± 4.9% vs. 27.3% ± 6.4%, *p* = 0.056). Bland–Altman analysis indicated minimal mean difference of 0.4% with moderate limits of agreement (LoA) spanning − 7.24% to + 8.07%, with good agreement for values of up to 35% (mean difference 0.1%, LoA: − 5.38% to + 5.23%). Fully automated synthetic ECV offers a blood-free proof-of-concept for large-scale post-processing, supporting consistent and efficient assessment of myocardial fibrosis in research settings, pending further validation for clinical use at higher ECV ranges.

**Supplementary Information:**

The online version contains supplementary material available at 10.1038/s41598-026-43624-3.

## Introduction

Myocardial fibrosis, whether localized or diffuse, is highly prevalent and associated with impaired clinical outcomes across many chronic cardiac conditions^1^. Presently, cardiac magnetic resonance (CMR) mapping techniques serve as core modalities for non-invasively assessing myocardial diffuse fibrosis and are emerging as biomarkers in image-based diagnostics^1–3^. While endomyocardial biopsy is still considered gold standard for fibrosis quantification, non-invasive CMR mapping techniques offer some significant advantages including great reproducibility, low accompanying risk, and the ability to provide additional information of the whole heart and adjacent structures^1,4^.

Over the past decades, developments such as short, one-breath hold mapping technology have made it possible to routinely obtain parametric maps and calculate the extracellular volume (ECV), based on the assumption of an equilibrium between the contrast concentration in the blood and the myocardial extracellular space, in a diverse group of patients^3,5^. For accurate assessment, the 2017 Society for Cardiovascular Magnetic Resonance (SCMR) Consensus Statement on parametric mapping recommends hematocrit (HCT) sampling within 24 h of the CMR scan^3^. Despite increasing use of CMR, not all providers have the infrastructural ability to routinely obtain hematocrit on-site. Additionally, hematocrit is subject to marked intraindividual fluctuations and even susceptible to changes as subtle as body position, with variations of approximately 8%^6^. To overcome these limitations and to facilitate faster CMR post-processing times, several groups have developed approaches to derive a “synthetic” HCT from T1 times in the blood pool, directly reflecting the HCT in the heart chamber during the supine scan^2,7,8^. Subsequently, the efficacy and reproducibility to calculate a “synthetic ECV” using only CMR-based parameters has been demonstrated, omitting the need for a periprocedural blood draw^7–10^.

However, aside from HCT sampling, conventional ECV measurement still involves a multi-step process of image acquisition, motion correction and manually contoured pre- and post-contrast mapping, leaving room for errors and interobserver variability at each step throughout the process. Further, practical use in the clinical setting still demands specialized hardware, software, trained staff, and protocols for data acquisition and analysis, which have yet to be fully standardized^3^. Thus, results vary depending on CMR vendor, site-dependent calibration of the hardware and sequence settings, but also simply intra- and interobserver variability, complicating unified interpretation^3^. Artificial intelligence (AI) and machine learning models offer the simplification of workflows and support automated analysis of complex spatio-temporal data. Namely, convolutional neural networks have aided in the fully automated segmentation of cardiovascular structures in various CMR applications^11–14^.

A fully automated synthetic ECV without the need of blood sampling and manual image processing could enhance the accessibility of ECV in routine clinical CMR and improve clinical decision-making^7,8^. The automatic generation of real-time synthetic ECV values during scans could be implemented across various CMR vendor platforms and medical centers, broadening the routine assessment of ECV in patients undergoing contrast-enhanced CMR^8^.

Thus, our goal was to assess the feasibility of automatically measuring blood-draw-free synthetic ECV using machine learning and compare its accuracy to the conventional ECV, which is manually contoured and based on lab-derived HCT values. In this study, we developed and tested a U-net-based approach for a fully automated machine learning-based assessment of synthetic ECV across different field strengths in a large routine clinical cohort.

## Methods

### Study design and participants

This retrospective investigation is a secondary analysis of 1101 study subjects who had previously undergone clinical CMR scans at the German Heart Center Berlin (now German Heart Center Charité, part of Charité—Universitätsmedizin Berlin) from August 2014 through November 2020.

The study complies with the declaration of Helsinki and all methods were performed in accordance with relevant guidelines and regulations. Ethical clearance for this research was acquired from the institutional ethics committee (Ethikkommission der Charité—Universitätsmedizin Berlin, Ethics number: EA2/073/21, Amendment number: 1), which approved informed consent waived from all involved participants, given the retrospective nature of this study.

Inclusion criteria for this cohort, initially described by Chen et al.^8^ and thus only briefly covered here, comprised the availability of both pre- and post-contrast T1 mapping data, as well as point-of-care or laboratory HCT measurements obtained within 24 h of the CMR scans. Exclusion criteria included severely impaired image quality, such as that caused by arrhythmia or breathing artifacts, as well as incomplete imaging or laboratory data.

**Fig. 1:**
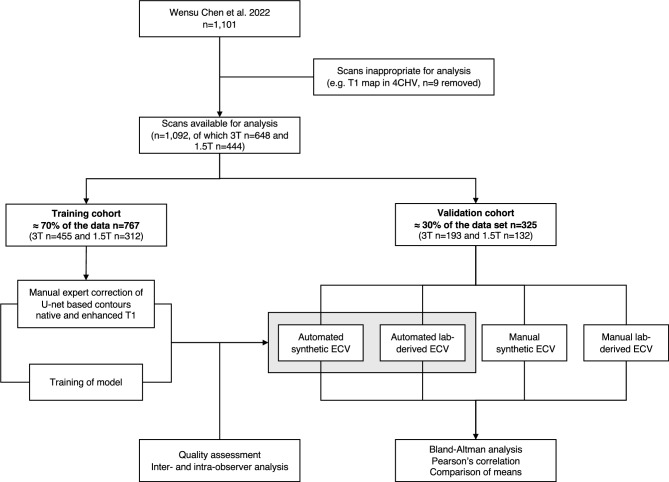
Overview of the study design and workflow. The study encompassed a total of 1092 scans, which were randomly divided into training and validation cohorts in a 70:30 ratio, respective for each field strength. Images in the training cohort underwent manual segmentation by a CMR expert reader and were subsequently entered into a U-net-based machine learning algorithm, tailored for native and contrast-enhanced T1 maps. This algorithm was then applied to the validation cohort, whose image and segmentation quality were evaluated by two independent CMR experts. ECV values calculated from the extracted T1 values and either laboratory-based or synthetic hematocrit (HCT) of the validation cohort were compared using Bland–Altman analysis, Pearson correlation, and paired Student’s t-tests.

For this study, images from an additional 9 scans had to be excluded prior to analysis because they were either not completely available at the time of the study or were not suitable for the current analysis (e.g., T1 map performed in a 4-chamber view slice). Ultimately, a total of 1092 CMR studies were included in the analysis. Of these, 648 subjects underwent CMR scans at a magnetic field strength of 3.0 Tesla (T), while 444 subjects were imaged at a magnetic field strength of 1.5 T (Fig. 1). Additionally, relevant clinical data, including demographic information such as age, sex, and referral diagnoses were available^8^.

Patients were randomly divided into a training and a validation cohort in a 70:30 ratio, respective for each field strength, as seen in Fig. 1. This resulted in 767 patients in the training group (3 T n = 455 and 1.5 T n = 312) and the remaining 325 in the validation group (3 T n = 193 and 1.5 T n = 132).

### CMR protocol

Among the study participants, 648 individuals underwent CMR imaging utilizing a clinical 3 T MRI scanner (Ingenia, Philips Healthcare, Best, The Netherlands), equipped with both anterior and built-in posterior coil arrays, employing up to 30 coil elements. The remaining 444 subjects were examined utilizing a 1.5 T MRI system (Achieva, Philips Healthcare, Best, The Netherlands) equipped with a cardiac 5-element phased array coil. No relevant hardware or software upgrades affected the results during the entire study period. All imaging was performed according to the recommendations of the Society of Cardiac Magnetic Resonance (SCMR)^15^. Cine images were acquired with a retrospectively gated cine-CMR approach, encompassing short-axis, vertical long-axis, and horizontal long-axis orientations, all utilizing a balanced steady-state free precession sequence. All patients presented with a clinical indication for CMR and underwent a standard clinical scanning protocol with late gadolinium imaging. Patients received a standardized dose of 0.15 mmol/kg of gadolinium-based contrast agent (Gadobutrol 1.0 mmol/mL, Gadovist, Bayer AG, Leverkusen, Germany). Native and 15-min post-contrast T1 mapping was carried out using a modified Look-Locker (MOLLI) sequence with a 5s(3s)3s-scheme, which has been described and tested by Kellman and Hansen^16^. For this, the default T1 mapping protocol (MOLLI 3s(3s)3s) provided by the vendor was employed, having identical settings for most relevant parameters like inversion time scheme, spatial resolution and scan duration at both field strengths. Identical settings before- and after CA were used in order to keep the potential propagation of systematic errors of T1 measurements into ECV (derived from native and contrast-enhanced T1 maps) small. The routine imaging parameters used in this cohort at our center have been previously described^8,16^.

### Conventional clinical CMR analysis

Clinical image analysis was conducted according to SCMR practice guidelines, utilizing commercially available postprocessing software (Philips Intellispace Portal, Philips Medical Systems Nederland BV, Best, The Netherlands), as previously described^3,8^. Manual delineation of left ventricular endocardial contours was performed on short-axis cine images at both end-diastole and end-systole, excluding trabeculation and papillary muscles. Left ventricular (LV) outflow tract was included in the LV cavity. LV function was assessed through measurements of ejection fraction, end-diastolic volume, end-systolic volume, stroke volume, and cardiac output. Additionally, body surface area-indexed end-diastolic myocardial left ventricular mass was assessed.

Native and post-contrast MOLLI images were subject to correction for in-plane motion using automatic motion correction algorithms provided by the post-processing software. This was achieved using elastic image registration based on the raw image data, yielding a motion-corrected T1 map. Following the guidelines, regions of interest were conservatively drawn in the intraventricular septum on the native T1 map, as well as in the left- and right-ventricular blood pools on the basal-ventricular short axis and subsequently transposed to the corresponding post-contrast maps^3^. A flow chart visualizing the separate steps of the processes of both the conventional and automated contouring is provided in the Supplementary Material.

Conventional ECV was calculated with the following equation utilizing native and post-contrast T1 relaxation times in conjunction with HCT values collected within 24 h of CMR:

1 $$\text{ECV }= \left(1-\mathrm{HCT}\right)\times \frac{\frac{1}{\mathrm{T}1\text{myo post}}- \frac{1}{\mathrm{T}1\text{myo native}})}{(\frac{1}{\mathrm{T}1\text{blood post}}-\frac{1}{\mathrm{T}1\text{blood native}} )}$$

### U-net training and iterative learning for automated assessment

Scanner-generated T1 maps without motion-correction were processed in a prototype to collaboratively develop machine-learning applications, which was used for manual contouring, refinement, and supervision of machine learning-based segmentation in the training group. A total of 767 native and post contrast images underwent careful review and manual segmentation by a clinical CMR expert.

**Fig. 2:**
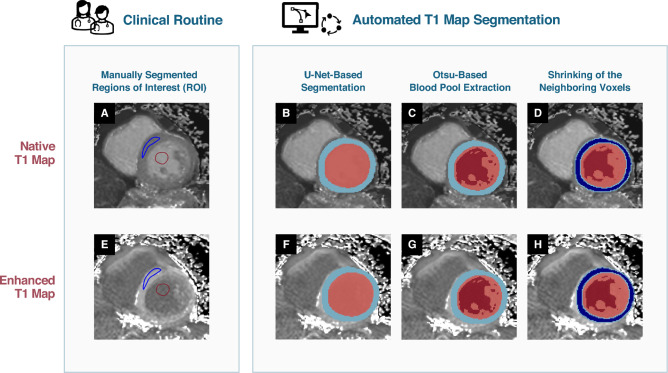
Visualization of the manual (**A + E**) and the automated (**B-D + F–H**) segmentation process in native T1 maps (**A–D**) and contrast-enhanced T1 maps (**E–H**). When manually assessed, the region of interest (ROI) was drawn in the septum and the blood pool as shown in (**A + E**) to measure T1 values relaxation times. The automated algorithm initially identified epi- and endocardial borders (**B + F**), papillary muscles were then removed from the blood pool using Otsu’s thresholding (**C + G**), and contours were subsequently refined by a one-voxel shrinkage to mitigate partial volume effects and motion artifacts (**D + H**).

Specifically, as depicted in Fig. 2, all images in the training data set were manually contoured along the full epicardial and endocardial borders of the left and the endocardial outline of the right ventricle (RV), respectively. Based on these contours, two-dimensional models for pre- and post-contrast T1 maps were trained using the nn-Unet framework (version 2.2) for precise delineation of cardiac structures^17^. The nn-Unet framework extracts a fingerprint of the dataset and configures the hyperparameters according to data characteristics automatically. For both contrasts, all datasets were used to train a separate model, with each a patch size of 256 × 256, a voxel spacing of 1.17 mm^2^ and a batch size of 49. Data was normalized using z-score normalization. The default data augmentation of the nn-Unet framework was applied.

In accordance with current consensus recommendations, morphologic erosion with a kernel size of 3 × 3 voxels, was applied to shrink margins from the endo- and epicardial border. Specifically, one voxel was removed from alongside both margins, leveraging a conservative approach especially in images with small intraventricular septum diameter, thus reducing the influence of partial volume effects in the setting of motion uncorrected images. Otsu’s thresholding method was employed for differentiation between the blood pool and adjacent structures like papillary muscles with a 3 × 3 voxel erosion applied in the same manner as with the myocardium.

Images were revised and then reincorporated into the training algorithm, fostering an iterative learning process of the model. This “expert in the loop” approach ensured accuracy of the generated results that were subsequently fed into the learning algorithm.

Ultimately, the supervised machine learning-algorithm was employed on the remaining 329 study subjects for validation. Median T1 relaxation times were extracted from the respective segmented regions of native and contrast-enhanced images.

### Quantitative and qualitative assessment of automated contours

Objective quantification of contour agreement was performed by manually segmenting the 325 validation cases to compare full-slice with full-slice using established geometric similarity metrics. Spatial overlap between automated and expert manual full-slice segmentations of the myocardium and blood pool was assessed using the Dice similarity coefficient, while boundary agreement of endo- and epicardial contours was evaluated using Hausdorff distances. All values were reported as mean ± standard deviation with corresponding 95% confidence intervals.

For qualitative assessment, a set of 100 native and contrast-enhanced pairs from the validation group was randomly selected and evaluated by two independent CMR expert reviewers. The reviewers used a numeric slider (0–100) on a 5-point categorical scale (“uninterpretable,” “poor,” “acceptable,” “good,” and “excellent”), adapted from Zhang et al.^18^ Segmentation quality was graded for adherence to anatomical structures, specificity for the segmented structures and interpretability. The comprehensive criteria used to rate image and segmentation quality can be found in the supplemental material.

### Calculation of synthetic ECV

Chen et al. previously published a total of eight distinct regression models to predict synthetic HCT, generated to account for different combinations of sex, magnetic field strength, and blood pool measurement, the reproducibility of which have been externally validated by Biso et al.^8,9^. In this study, the respective models, specific for sex and field strength, were employed for the computation of the synthetic HCT values, due to their improved performance over the general models, especially in the setting of anemia^8^.

Median values for both native and contrast-enhanced T1 relaxation times were obtained from automated myocardial and blood pool contours. These values were then used for synthetic HCT generation and ECV calculation, using the following formula:

2 $$\text{synthetic ECV }= \left(1-\text{synthetic HCT}\right)\times \frac{\frac{1}{\mathrm{T}1\text{myo post}}- \frac{1}{\mathrm{T}1\text{myo native}})}{(\frac{1}{\mathrm{T}1\text{blood post}}-\frac{1}{\mathrm{T}1\text{blood native}} )}$$

### Statistical analysis

Descriptive data were presented as mean and standard deviation for continuous variables, and as count and percentages for categorical variables with 95% confidence interval indicated.

Bland–Altman analysis, paired student’s t-test, and Pearson correlation were conducted to assess the level of agreement between conventional and synthetic ECV. Additionally, paired Student’s t-tests and Pearson correlation were utilized to compare the individual components used in ECV calculation. Diagnostic agreement between conventional and fully automated synthetic ECV was assessed using threshold-based comparisons. Two cut-offs were tested: first, 30% for both methods; and second, 29.5% for synthetic ECV and 30% for conventional ECV, respectively, in line with the threshold adaptation proposed by Chen et al.^8^ McNemar’s test was used to evaluate statistical differences in classification. These analyses were repeated for full-slice to full-slice comparison, presented in the Supplementary Material.

Intra- and interobserver agreement regarding image and segmentation quality ratings were analyzed using intraclass correlation coefficients (ICC): ICC (2,k) for interobserver variability and ICC(2,1) for intraobserver variability.

All statistical analyses were conducted using R version 4.3.0. The threshold for statistical significance was set at an unadjusted two-sided *p*-value of less than 0.05.

## Results

### Descriptive statistics of studied cohort

In total, 1092 subjects with a broad spectrum of clinical indications for contrast-enhanced CMR were included in the study, encompassing 39% females with an average age of 52 ± 17 years (mean ± standard deviation, Table 1)^8^. Both the training and validation cohorts for each field strength were well matched in terms of demographics, cardiac dimensions, and function. The only statistically significant difference observed was a higher conventional HCT in the 3 T validation group than in the 3 T training group (43.7 ± 4.8% vs. 42.7 ± 5.4%; *p* = 0.034). All results presented hereafter are specific to the validation group.

**Table 1:** Cohort characteristics.

			1.5 Tesla		3 Tesla	
Characteristics	n	All patients n = 1092^*a*^	Training n = 312^*a*^	Validation n = 132^*a*^	Training n = 455^*a*^	Validation n = 193^*a*^
Age (years)	1092	52 ± 17 [51, 53]	50 ± 17 [48, 52]	51 ± 16 [48, 54]	52 ± 17 [51, 54]	54 ± 17 [52, 57]
Male	1092	664 (61%) [58%, 64%]	187 (60%) [54%, 65%]	76 (58%) [49%, 66%]	282 (62%) [57%, 66%]	119 (62%) [54%, 68%]
BSA (m2)	1079	1.98 ± 0.24 [2.0, 2.0]	1.95 ± 0.24 [1.9, 2.0]	1.95 ± 0.22 [1.9, 2.0]	1.99 ± 0.25 [2.0, 2.0]	2.01 ± 0.24 [2.0, 2.0]
LVEDD (mm)	900	54 ± 8 [54, 55]	55 ± 8 [54, 56]	55 ± 9 [53, 57]	54 ± 8 [53, 55]	55 ± 7 [52, 57]
LVEDV (ml)	1088	169 ± 67 [165, 173]	172 ± 67 [165, 180]	181 ± 77 [168, 194]	167 ± 69 [160, 173]	160 ± 55 [152, 168]
LVEF (%)	1088	54 ± 13 [53, 54]	53 ± 13 [52, 55]	51 ± 15 [49, 54]	54 ± 13 [53, 56]	54 ± 12 [52, 56]
IVSd (mm)	906	11.8 ± 5.1 [11, 12]	11.5 ± 4.9 [11, 12]	10.9 ± 3.0 [10, 11]	12.1 ± 5.6 [12, 13]	12.6 ± 3.5 [11. 14]
LVPWd (mm)	860	7.83 ± 2.14 [7.7, 8.0]	7.82 ± 2.14 [7.6, 8.1]	7.48 ± 1.72 [7.2, 7.8]	7.89 ± 2.20 [7.7, 8.1]	8.17 ± 2.58 [7.3, 9.1]
LA (mm2)	900	23 ± 7 [22, 23]	23 ± 7 [22, 24]	24 ± 8 [23, 26]	23 ± 7 [22, 23]	23 ± 7 [21, 25]
Hematocrit (%)	1092	42.6 ± 5.4 [42, 43]	42.2 ± 5.4 [42, 43]	41.7 ± 6.2 [41, 43]	42.7 ± 5.4 [42, 43]	43.7 ± 4.8 [43, 44]
ECV (%)	1092	27.2 ± 6.0 [27, 28]	27.4 ± 5.3 [27, 28]	28.4 ± 7.5 [27, 30]	27.0 ± 6.1 [26, 28]	26.5 ± 5.8 [26, 27]
Native T1 times (ms)	1092	1175 ± 133 [1167, 1183]	1031 ± 64 (1024, 1038)	1036 ± 60 (1025, 1046)	1274 ± 62 (1268, 1279)	1270 ± 60 (1262, 1279)

^*a*^Mean ± SD; n (%); [95% CI].

BSA, body surface area; ECV, myocardial extracellular volume; LA, left atrial diameter; IVSd, intraventricular septum diameter; LVEDD, left ventricular end diastolic diameter; LVEDV, left ventricular end diastolic volume; LVEF, left ventricular ejection fraction; LVPWd, left ventricular posterior wall diameter.

### Automatically assessed T1 values

Differences between clinically measured T1 values based on varying sex, field strength and heart chamber have previously been explored by our group^8^. Comprehensive results on the automatically extracted T1 times of each individual component used for automated ECV calculation are displayed in the Supplementary Material.

#### Native myocardial T1 relaxation times

At 1.5 T, the native T1 relaxation times derived from automatically contoured slices demonstrated a strong correlation with those obtained via manual contouring methods, supported by a Pearson correlation coefficient of r = 0.97 (*p* < 0.001). Statistically significant differences were observed in the mean native T1 relaxation times, with automatic contouring yielding lower values compared to manual contouring, which measured (1030 ± 60 ms vs. 1036 ± 60 ms; *p* < 0.001).

At 3 T, native T1 relaxation times from automated myocardial contours showed a strong correlation with manually drawn contouring, with a Pearson correlation of r = 0.95 (*p* < 0.001), whilst exhibiting significantly lower native T1 relaxation times than the manually contoured regions of interest (ROI) (1262 ± 62 ms vs. 1270 ± 61 ms, *p* < 0.001).

#### Contrast-enhanced myocardial T1 relaxation times

At 1.5 T, there was no significant difference in contrast-enhanced T1 relaxation times derived from automatic contouring of myocardium and those measured manually, with no significant difference noted (444 ± 52 ms vs. 449 ± 51 ms, *p* = 0.294). Both methods demonstrated a strong correlation (r = 0.93, *p* < 0.001).

At 3 T, automated myocardial contours produced post-contrast T1 relaxation times that were only slightly but statistically significantly higher compared to manually drawn ROI contouring (515 ± 53 ms vs. 514 ± 60 ms; *p* = 0.003), while maintaining a strong correlation (r = 0.94, *p* < 0.001).

#### Native blood pool T1 relaxation times

Otsu’s thresholding-based assessment of native LV blood pool relaxation times yielded significantly lower automated values compared to conventionally derived measurements. Specifically, at 1.5 T, the Otsu-based native T1 times for the blood pool were 1546 ± 115 ms, while the manually measured values were 1559 ± 121 ms (*p* < 0.001). Similarly, at 3 T, the Otsu-based native T1 times were 1824 ± 91 ms, in contrast to the manually measured values of 1839 ± 90 ms (*p* < 0.001). Otsu-based and manually-contoured native T1 relaxation times showed a Pearson correlation of r = 0.97 (*p* < 0.001) at 1.5 T and of r = 0.95 (*p* < 0.001) at 3 T, respectively.

#### Contrast-enhanced blood pool T1 relaxation times

Otsu’s thresholding-based assessment of contrast-enhanced LV blood pool T1 relaxation times indicated significantly higher automated values than those obtained manually. At 1.5 T, the Otsu-based post-contrast T1 times for the blood pool were 312 ms ± 52 ms, compared to manually measured values of 311 ms ± 58 ms (*p* < 0.354). At 3 T, the Otsu-based post-contrast T1 times were 333 ms ± 46 ms, while the manually measured values were 330 ms ± 47 ms (*p* = 0.010). In congruence with native T1 relaxation times, automated and manual contrast-enhanced blood pool T1 relaxation times correlated strongly (r = 0.96, *p* < 0.001 at 1.5 T and r = 0.97, *p* < 0.001 at 3 T).

### Synthetic, automated ECV vs. conventional, manually contoured ECV

**Fig. 3:**
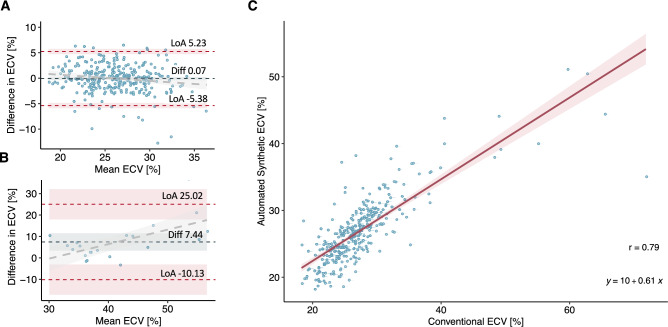
The Bland–Altman plots (**A + B**) illustrate the agreement between fully automated synthetic ECV and conventional ECV, with the dark blue lines representing the mean difference (bias) between the methods, while the red dashed lines indicate the limits of agreement (95% confidence interval of the differences between the measured values). The grey dashed lines reflect the increase in difference between the two methods for increasing ECV values. Panel (**C**) displays the strong correlation between automated synthetic ECV and conventionally measured ECV, with the corresponding linear regression equation (regression line in red) and Pearson correlation coefficient (r) provided. LoA = Limits of agreement. Diff = Mean difference.

**Fig. 4:**
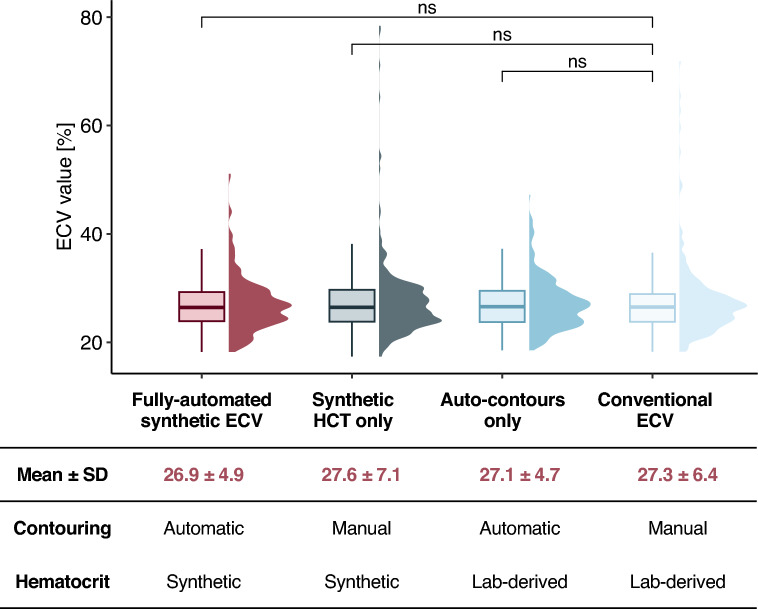
The depicted boxplots demonstrate that fully automated synthetic ECV (dark red) exhibited no significant difference compared to manually measured conventional ECV (light blue) when tested with a two-sided paired t-test. Similarly, no significant differences were observed for synthetic ECV measured manually (dark grey), nor for ECV from automated contouring using lab-derived HCT (blue). The corresponding values are presented as mean ± standard deviation in the table. ECV = Myocardial extracellular volume. HCT = Hematocrit. SD = Standard deviation. NS = Statistically not significant.

A strong correlation was observed between synthetic automated ECV and conventionally measured ECV (r = 0.79, *p* < 0.001, Fig. 3C). Notably, no statistically significant mean differences were detected between the synthetic automated ECV and the conventional manually contoured ECV numerically (26.9 ± 4.9% vs. 27.3 ± 6.4%) without reaching statistical significance (*p* = 0.056, Fig. 4).

This translates to a statistically non-significant mean difference of 0.4% with a 95% confidence interval (CI) of − 0.01% to 0.84% in the Bland–Altman analysis between the automated synthetic ECV and conventional assessment (*p* = 0.056). The limits of agreement (LoA) spanned from − 7.2% (95% CI − 7.97% to − 6.50%) to 8.1% (95% CI 7.33% to 8.80%) indicating a moderate degree of discrepancy across the measurements (Supplementary Material). Bland–Altman analysis suggested a pattern of increasing variation at higher measurement values. For ECV values ≤ 35% (Fig. 3A), the automated synthetic ECV showed very good agreement with conventionally measured ECV, exhibiting a mean difference of 0.1% (95% CI − 0.38 to 0.23%) and LoA spanning from -5.4% (95% CI − 5.91 to − 4.85%) to 5.2% (95% CI 4.71 to 5.76%). However, for values exceeding 35% (Fig. 3B), agreement was significantly poorer, characterized by a mean difference of 7.4% (95% CI 3.36 to 11.53%) and LoA ranging from − 10.1% (95% CI − 17.20 to − 3.06%) to 25.0% (95% CI 17.95 to 32.09%).

When assessing the individual components used for the calculation of synthetic automated ECV, manually-contoured synthetic ECV derived from ROI contours and synthetic HCT (27.6% ± 7.1%) did not differ significantly from conventional ECV (27.3% ± 6.4%, *p* = 0.102). Similarly, ECV derived only from automated contours, while using laboratory-derived hematocrit, was on average 0.2% lower (27.1% ± 4.7%) than conventional ECV, exhibiting no statistically significant difference (*p* = 0.738).

Classification analysis using a threshold of 30% for both conventional and fully automated synthetic ECV demonstrated concordant classification in 87.7% of cases. Specifically, 241 individuals (74.2%) were classified as negative and 44 (13.5%) as positive by both methods. Discrepancies were observed in 40 cases, with 18 (5.5%) identified as false negatives and 22 (6.8%) as false positives by the fully automated synthetic method. McNemar’s test showed no statistically significant difference in classification between the two approaches (*p* = 0.635).

Applying a lower threshold of 29.5% to synthetic ECV, as proposed by Chen et al., while maintaining the conventional threshold of 30%, resulted in concordant classification in 86.5% of cases^8^. In this setting, 233 individuals (71.7%) were classified as negative and 48 (14.8%) as positive by both methods. The number of false negatives decreased to 14 (4.3%), while false positives increased to 30 (9.2%). McNemar’s test indicated a statistically significant difference in classification for this threshold combination (*p* = 0.024). The corresponding contingency tables are provided in the Supplementary Material (Table 2).

**Table 2:** ECV value based on segmentation approach and hematocrit sampling method.

Field strength	Label	Contouring method	Hematocrit method	ECV value^*a*^	*p*-value (vs. conventional ECV)^b^
1.5T	Conventional ECV	Manual	Laboratory	28.6 ± 7.1 (27.4–29.9)	–
	Automated ECV	Automated	Laboratory	28.7 ± 4.7 (27.9–29.5)	0.929
	Manual Synthetic ECV	Manual	Synthetic	28.7 ± 7.8 (27.3–26.6)	0.778
	Fully Automated Synthetic ECV	Automated	Synthetic	28.4 ± 4.9 (27.5–29.2)	0.537
3T	Conventional ECV	Manual	Laboratory	26.5 ± 5.8 (25.7–27.4)	–
	Automated ECV	Automated	Laboratory	26.0 ± 4.4 (25.4–26.7)	**0.042**
	Manual Synthetic ECV	Manual	Synthetic	26.8 ± 6.5 (25.9–27.7)	0.063
	Fully Automated Synthetic ECV	Automated	Synthetic	26.0 ± 4.7 (25.3–26.6)	**0.021**
All	Conventional ECV	Manual	Laboratory	27.3 ± 6.4 (26.6–28.0)	–
	Automated ECV	Automated	Laboratory	27.1 ± 4.7 (26.6–27.6)	0.246
	Manual Synthetic ECV	Manual	Synthetic	27.6 ± 7.1 (26.7–28.3)	0.102
	Fully Automated Synthetic ECV	Automated	Synthetic	26.9 ± 4.9 (26.4–27.5)	0.056

“Significant values are in [bold]”

^a^reported in mean ± SD.

^b^paired t-test when compared to Conventional ECV as reference, within respective field strength (1.5T vs. 1.5T, 3T vs. 3T, and all vs. all).

### Assessment of image quality and performance of automated contours

**Fig. 5:**
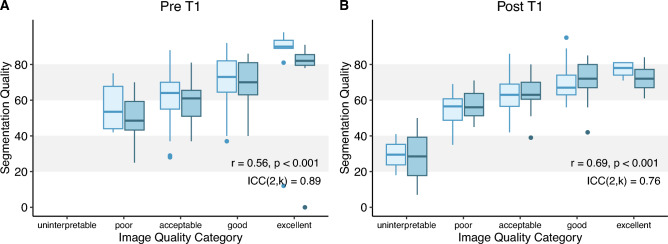
Association between image quality and segmentation quality for native (**A**) and contrast-enhanced (**B**) maps assessed by two independent, CMR-experienced raters. A numeric slider (0–100) on a 5-point categorical scale ("uninterpretable," "poor," "acceptable," "good," and “excellent”) was used to grade the model’s performance. Segmentation quality overall was good (mean 66 ± SD 16 for native and 64 ± 11 for contrast-enhanced maps) and improved with increasing image quality (r = 0.56, *p* < 0.001 for native and r = 0.69, *p* < 0.001 for contrast-enhanced images). Intraclass correlation coefficient (ICC) was used to assess interobserver agreement with regards to segmentation quality and is provided in the figure.

Analysis of the randomly selected T1 mapping pairs (Fig. 5) indicated generally acceptable to good image quality, with native T1 images achieving a mean score of 59 ± 16 and post-contrast T1 images receiving a mean score of 54 ± 16. The automated algorithm successfully delineated the relevant cardiac structures in the 100 images pairs, with only 1 native and 1 contrast-enhanced segmentation rated as uninterpretable. Furthermore, only 3 native segmentations were deemed poor, with all other segmentations reaching a quality of acceptable or higher. In fact, 62% of native and 66% of contrast-enhanced segmentations were rated as good or excellent. Segmentation quality improved significantly with better image quality, with r = 0.56, *p* < 0.001 for native images and r = 0.69, *p* < 0.001 for contrast-enhanced images, respectively.

Regarding image quality of the maps, intraobserver variability for native and contrast-enhanced images for operator 1 were ICC = 0.76 and ICC = 0.80, as well as for operator 2 ICC = 0.81 and ICC = 0.85, respectively. Corresponding interobserver variability was ICC = 0.86 for native T1 maps and ICC = 0.84 for contrast-enhanced T1 maps. For segmentation quality, intraobserver variability was ICC = 0.70 and ICC = 0.80 in native images and ICC = 0.72 and ICC = 0.80 in contrast-enhanced images for operators 1 and 2, respectively. Segmentation interobserver variability was ICC = 0.89 for native T1 maps and ICC = 0.76 for contrast-enhanced T1 maps.

## Discussion

### Summary of key findings

In this study, we introduced an integrated approach combining machine learning technology as a proof-of-concept for automated segmentation of T1 maps with a blood draw-free estimation of HCT for the calculation of myocardial ECV. Our aim was to provide an automated, fast, reproducible, and scalable approach for ECV calculation. To the best of our knowledge, this study represents the first evaluation of their combined performance.

In summary, automated synthetic ECV yielded quantifications that (a) showed a highly significant correlation with conventional ECV at both 1.5 T and 3 T, and (b) showed good agreement with conventional ECV in the clinically critical ranges up to 35% with a decreased performance in higher ranges. The results of our analysis indicate that a combination of the presented AI deep learning network and the previously established field strength and gender-specific regression model by Chen et al.^8^, may pose a practical and effective approach to retrospectively batch-quantify ECV or distinguishing between normal and diseased conditions for research purposes. While further refinement is needed to fully automate clinical practice, this method could effectively replace both on-site blood draw and manual contouring with AI-driven post-processing in the research setting.

*The developed application for the fully automated calculation of synthetic ECV from pre- andpost-contrast T1 maps has been made open-source to be used in the research setting. The code* *(*https://git-ext.charite.de/diamo/syntheticecv*) and the model(*https://zenodo.org/records/13842603*) are publicly available*.^19^

Adopting the nn-Unet framework for image segmentation, our data showed a high degree of correlation (Pearson r ranging from 0.93 to 0.96, with *p*-values < 0.001) between automatically contoured and conventionally assessed T1 relaxation times, consistent with findings from prior studies that utilized various approaches for image segmentation^20–23^. Fahmy et al. trained a fully convolutional neural network on T1-weighted images of 210 patients and found strong correlation in per-patient analyses (r = 0.82)^21^. Similarly, Farrag et al. used a U-net-based approach for segmentation of native and post-contrast T1 maps^23^. Puyol-Antón et al. employed a PHiSeg network in a large UK biobank data set, showing good reproducibility between automatic and manual contours with a dice score of 0.84, which was similar to the range of manually drawn inter- and intraobserver variabilities in their assessment^20^. In conjunction with findings from these studies, our results demonstrate that automated segmentation of parametric T1 maps is feasible and accurate, holding the potential to shorten post-processing times required for ECV assessment.

We did observe small but statistically significant differences between automatic and manual T1 values, with the magnitude of these differences partly depending on the respective field strength and contrast enhancement. In line with the image and segmentation quality analysis performed (Fig. 5), such discrepancies may be attributable to the influence of images with lower quality or uneven myocardial morphology from motion uncorrected images, which, despite shrinking the myocardial area at the respective endo- and epicardial borders, may still have resulted in partial volume overlap. It is also worth noting that the methodological bias arising from comparing the manually contoured septal region to the automatically derived entire myocardial slice inherently leads to a certain degree of differences seen in T1 values, as septal T1 values are typically 50 to 100 ms higher than those of the lateral wall in native T1 acquisitions, with the reverse pattern seen in post-contrast imaging^24^. Lastly, pathologies such as papillary muscle fibrosis, for instance, frequently observed in conditions like mitral valve prolapse, may present elevated T1 relaxation times, potentially exceeding the binary threshold established by Otsu’s method^25^. To address this, we excluded neighboring voxels in the blood pool, which also served to mitigate the influence of potentially altered T1 relaxation times caused by close turbulent flow.

While segmentation effects may propagate into ECV measurement, our analysis found no statistically significant differences between the conventional and fully automated synthetic methods, with a mean difference of only 0.4%, a range considerably low given known clinical and interobserver variability. In a previous study of healthy volunteers, Aus dem Siepen and colleagues observed no significant change in ECV over a period of approximately three years, with a coefficient of variation of 3.5%^26^. For conventional ECV, Yingchoncharoen et al. reported an interobserver variability of around 2% in manually performed measurements^27^.

It is important to note that in our study, the scope of variability seen in Bland–Altman analysis between automated synthetic ECV and conventional ECV depended on the ECV range. Specifically, agreement was particularly strong for values within the range of 0–35%, which includes the critical clinical threshold that is usually based on the upper limits of normal and typically found around 30%^28,29^. However, Bland–Altman analysis also revealed unexpectedly large discrepancies at higher ECV values. This discrepancy is particularly evident in Figs. 3b and 4, where synthetic ECV and conventional ECV data points in the high ECV ranges were significantly underestimated when using T1 relaxation times derived from automated contouring.

Several factors could account for this. For instance, a visual review of image quality and contouring performance (see Supplementary Material) revealed that lower image quality, such as uncorrected motion artifacts or poor contrast differentiation between myocardium and blood (e.g. in amyloidosis cases) contributed to contouring errors in higher ECV ranges. In addition, model performance is likely compounded by the relatively small number of high-ECV cases available for model training and validation. In this cross-sectional cohort from a tertiary care center, ECV values above 35% represented only a minor proportion of the population (79 of 1092 cases; approximately 7.2%). This imbalance reflects a sparsity of training data in the higher ECV range, likely limiting the model’s ability to generalize robustly to myocardial tissue phenotypes typically occurring in these ranges. Further, the comparison between full-slice myocardial and septal measurements may mitigate a potentially locally elevated ECV seen in conditions with predominantly septal disease^30^. Previous studies found ECV values to be strongly associated with regional wall thickness and to vary within myocardial regions, as well the standard deviation of the measured ECV to be lower in larger ROI^30,31^. Similarly, in some cases regional foldover artifacts were identified as potential sources of error, which were included by the algorithm, despite (or because of) adequate preservation of myocardial segmentation (exemplary images can be found in the Supplementary Material). In response to visual artifacts such as those mentioned above or suspected regionally distributed disease, clinical users often visually adjust their ROI to a smaller, more specific area, typically in the septal basal myocardium, to ascertain the “true” ECV. This level of adaptability is lacking in our proposed algorithm and should be considered when interpreting the results.

While our algorithm for fully automated synthetic ECV may require further validation in higher ECV ranges (> 35%), it demonstrated reasonable correlation with conventional ECV measurements. Additionally, classification analysis with an adapted threshold of 29.5% showed robust accuracy with a false negative rate of below 5%, making the algorithm particularly suited for batch processing in research settings. While inline tools for the calculation of synthetic ECV have been introduced to post-processing software, the efficient analysis of large datasets without the need for hematocrit sampling nor manual contouring on a patient-by-patient basis—as it is provided by this algorithm—minimizes human input and may ultimately reduce variability and use of resources, making it particularly effective for environments where rapid processing of extensive datasets is essential. Sites wishing to apply this model are encouraged to validate it against their local T1 mapping protocols, scanner hardware, and patient populations, contributing to the ongoing refinement and calibration of the model across different sites. While further improvements are needed for clinical use, we believe that this application provides a valuable tool for large-scale research studies and retrospective analyses, improving the efficiency of myocardial fibrosis assessment in CMR.

### Limitations and future work

Several limitations of this study have to be acknowledged. First, to increase reproducibility, we trained the U-net on a large number of scans with varying image quality that were taken from two separate scanners with different field strengths. However, as the presented machine-learning algorithm was trained and tested in a single-center using only Philips scanners, the generalizability of the model is limited given the varying scanner configurations across vendors. Consequently, as for any other mapping parameter, the performance of this model may vary across sites, especially when applying other mapping sequences such as shortened modified Look-Locker inversion recovery (ShMOLLI) or saturation recovery single-shot acquisition (SASHA) and thus, will require local validation to confirm the suitability of local T1 mapping ranges^7,32,33^. While the ideal approach would be for each site to develop their own synthetic ECV regression model based on local data, Fent et al.’s synthetic ECV model has been compared with a locally derived model at a different site by Censi et al. who found that although the locally derived model offered better accuracy, both approaches proved effective^10,34^. Generally, for application of the publicly available tool at other vendors and sites, we propose a sample size of 15–20 healthy subjects for initial testing of the algorithm, as it is recommended by the SCMR for T1 mapping normal values^3^. Potential strategies for addressing issues could involve enabling the nn-Unet to automatically select model configurations based on the provided images instead of applying the hyperparameters from this study (provided in the Supplementary Material), with adjustments to the ECV cutoff thresholds as necessary. For Philips scanners employing the MOLLI sequence, we anticipate that the contouring should align reliably.

Second, contrary to the conventional analysis, T1 maps were not motion-corrected for neither model-training nor automated extraction of ECV values. Even though motion correction of T1 maps is considered state-of-the-art, not all post processing software available for either commercial or scientific use have this included as an automated feature. We acknowledge that omitting motion-correction may introduce bias, especially in cases with very thin myocardial wall, potentially resulting in areas of partial volume overlap. Yet, our results showed good agreement with conventional ECV. For optimized results, patient-friendly, short protocols and careful guidance during scanning should be employed, thereby increasing compliance to reduce breathing and motion artifacts to a minimum.

Third and evidently, regional ECV assessed using a septal region of interest is not equivalent to whole-slice ECV, which reflects the average signal across the entire myocardium between the endocardial and epicardial contours. This represents a methodological limitation, as the primary analysis compares two different measurement approaches. To address this and to isolate true algorithm-based deficiencies, we performed an additional full-slice-to-full-slice comparison between automated and manual ECV measurements in the Supplementary Material. In this region-matched analysis, agreement between methods was excellent, with a very high correlation (R = 0.98, *p* < 0.001) and minimal bias on Bland–Altman analysis (mean difference 0.21%, limits of agreement − 2.06 to 2.49%). These findings suggest that the discrepancies observed in the primary analysis are largely driven by differences in ROI selection (septal versus whole-slice) rather than by inherent limitations of the automated method itself. The complete analysis, including field-strength-specific results, is provided in the Supplementary Material.Fourth, Chen et al. have found and discussed synthetic ECV error to be higher in anemic than in nonanemic patients, which should be considered upon application to specific patient groups at risk for anemia^8^.

Future research should focus on external validation and cross-vendor testing, as well as improvement of automated synthetic ECV in higher ranges with datasets with a more balanced distribution. In addition, given the retrospective nature of our study, our findings should be validated in prospective cohort studies in the future. Lastly, the proposed approach could be integrated with complementary techniques, such as automated quality-control flags or artifact detection to support safer deployment, as well as advanced myocardial tissue characterization or data-driven deep learning methods aimed at quantifying myocardial tissue characteristics and mechanical properties, thereby enabling a more comprehensive assessment of myocardial pathology beyond conventional interpretation alone^35–37^.

## Conclusion

In conclusion, fully automated synthetic ECV presents a promising alternative to its conventional counterpart. While further refinement and external validation are necessary for clinical application, our results suggest that this automated method could offer substantial benefits in research applications. The automated assessment may not only eliminate the need for blood draws but also facilitate substantially easier (large-scale) post-processing. Consequently, the integration of the hereby publicly available application for fully automated synthetic ECV may enable a more consistent use of ECV as a powerful non-invasive biomarker in the research setting, thereby maximizing its potential of assessing diffuse myocardial fibrosis in CMR.

## Supplementary Information

Supplementary Information.

## Data Availability

The data underlying this article are available in the article and its online Supplementary Material, and additional data will be shared upon reasonable request to the corresponding author.
