# Supplementary material for: Towards fully automated synthetic ECV quantification: an open-access machine learning-based approach for fast blood draw-free CMR
Source: Sci Rep. 2026 Mar 10;16:8552. doi: 10.1038/s41598-026-43624-3 (PMC12976349; doi:10.1038/s41598-026-43624-3)
Supplement: Supplementary file 1 — Supplementary Information. [file 41598_2026_43624_MOESM1_ESM.docx]

**Supplements.**

Towards fully automated, synthetic ECV quantification – An open-access machine learning-based approach for fast and blood draw-free CMR

Table of Contents

[1 Methods 2](#_Toc208150103)

[1.1 Flow chart visualizing Post-processing and Motion correction 2](#_Toc208150104)

[1.2 Quality Rating 3](#_Toc208150105)

[1.2.1 Image Quality Rating 3](#_Toc208150106)

[1.2.2 Segmentation / Contouring Quality Rating 4](#_Toc208150107)

[2 Results 5](#_Toc208150108)

[2.1 T1 Relaxation times on which ECV calculations were based 5](#_Toc208150109)

[2.1.1 Myocardial Native T1 Values 5](#_Toc208150110)

[2.1.2 Myocardial Post T1 Values 6](#_Toc208150111)

[2.1.3 Blood Pool Native T1 Values 7](#_Toc208150112)

[2.1.4 Blood Pool Post T1 Values 8](#_Toc208150113)

[2.2 Voxel count on which ECV calculations were based 9](#_Toc208150114)

[2.3 Regression Models for lower ECV ranges 10](#_Toc208150115)

[2.3.1 Regression Model for ECV values <40% 10](#_Toc208150116)

[2.3.2 Regression Model for ECV values <50% 10](#_Toc208150117)

[2.4 Contingency tables for diagnostic agreement 11](#_Toc208150118)

[3 Discussion 12](#_Toc208150119)

[3.1 Outlier Assessment 12](#_Toc208150120)

[3.1.1 Bland-Altman plot for reference 12](#_Toc208150121)

[3.1.2 Exemplary visualization of native and contrast-enhanced T1 maps of outliers 13](#_Toc208150122)

# Methods

## Flow chart visualizing Post-processing and Motion correction

## Quality Rating

### Image Quality Rating

### Segmentation / Contouring Quality Rating

# Results

## T1 Relaxation times on which ECV calculations were based

### Myocardial Native T1 Values

| **Scanner** | **Contouring Method** | **Mean ± SD** | **Contouring Method** | **Mean ± SD** | **p-value*** |
| --- | --- | --- | --- | --- | --- |
| **1.5T** | Conventional ROI | 1036 ± 60 | Whole Myocardium | 1030 ± 59 | 0.019 |
|  |  |  | Shrunken Myocardium | 1028 ± 59 | <0.001 |
| **3T** | Conventional ROI | 1270 ± 61 | Whole Myocardium | 1262 ± 62 | <0.001 |
|  |  |  | Shrunken Myocardium | 1252 ± 62 | <0.001 |
| **Overall** | Conventional ROI | 1175 ± 130 | Whole Myocardium | 1168 ± 129 | <0.001 |
|  |  |  | Shrunken Myocardium | 1161 ± 126 | <0.001 |
| * Conventional ROI as Reference | | | | | |

### Myocardial Post T1 Values

| **Scanner** | **Contouring Method** | **Mean ± SD** | **Contouring Method** | **Mean ± SD** | **p-value*** |
| --- | --- | --- | --- | --- | --- |
| **1.5T** | Conventional ROI | 449 ± 51 | Whole Myocardium | 444 ± 52 | 0.003 |
|  |  |  | Shrunken Myocardium | 447 ± 52 | 0.294 |
| **3T** | Conventional ROI | 514 ± 60 | Whole Myocardium | 515 ± 53 | 0.310 |
|  |  |  | Shrunken Myocardium | 517 ± 54 | 0.036 |
| **Overall** | Conventional ROI | 487 ± 65 | Whole Myocardium | 486 ± 63 | 0.243 |
|  |  |  | Shrunken Myocardium | 488 ± 63 | 0.304 |
| * Conventional ROI as Reference | | | | | |

### Blood Pool Native T1 Values

| **Scanner** | **ROI Type** | **Mean Value ± SD** | **Comparison Group** | **Mean Value ± SD** | **p-value*** |
| --- | --- | --- | --- | --- | --- |
| **1.5T** | Conventional ROI | 1559 ± 121 | Automated (inkl. OTSU and 1-voxel) | 1546 ± 115 | <0.001 |
| **3T** | Conventional ROI | 1839 ± 90 | Automated (inkl. OTSU and 1-voxel)) | 1824 ± 91 | <0.001 |
| **Overall** | Conventional ROI | 1725 ± 172 | Automated (inkl. OTSU and 1-voxel)) | 1711 ± 170 | <0.001 |
| * Conventional ROI as Reference | | | | | |

### Blood Pool Post T1 Values

| **Scanner** | **ROI Type** | **Mean Value ± SD** | **Comparison Group** | **Mean Value ± SD** | **p-value*** |
| --- | --- | --- | --- | --- | --- |
| **1.5T** | Conventional ROI | 311 ± 58 | Automated (inkl. OTSU) | 312 ± 52 | 0.354 |
| **3T** | Conventional ROI | 330 ± 47 | Automated (inkl. OTSU) | 333 ± 46 | 0.010 |
| **Overall** | Conventional ROI | 323 ± 52 | Automated (inkl. OTSU) | 324 ± 50 | 0.015 |
| * Conventional ROI as Reference | | | | | |

## Voxel count on which ECV calculations were based

| Voxel count* | Native myocardium | Contrast-enhanced myocardium | Native blood pool | Contrast-enhanced blood pool |
| --- | --- | --- | --- | --- |
| Minimum | 43 | 6 | 186 | 84 |
| 1st Quartile | 333 | 182 | 739 | 840 |
| Median | 540 | 361 | 1041 | 1269 |
| Mean | 1683 | 1612 | 2831 | 3110 |
| 3rd Quartile | 3142 | 3038 | 4968 | 5367 |
| Maximum | 7196 | 8525 | 19577 | 18338 |
| Less than 20 voxels | 0 (0%) | 4 (1.23%) | 0 (0%) | 0 (0%) |

* Remaining voxels included in ECV calculation after automated contouring

## Regression Models for lower ECV ranges

### Regression Model for ECV values <40%

### Regression Model for ECV values <50%

## Contingency tables for diagnostic agreement

For both tests, a threshold of 30% was used for conventional ECV and the threshold as indicated for fully automated synthetic ECV (Synthetic ECV)

# Discussion

## Outlier Assessment

### Bland-Altman plot for reference

### Exemplary visualization of native and contrast-enhanced T1 maps of outliers


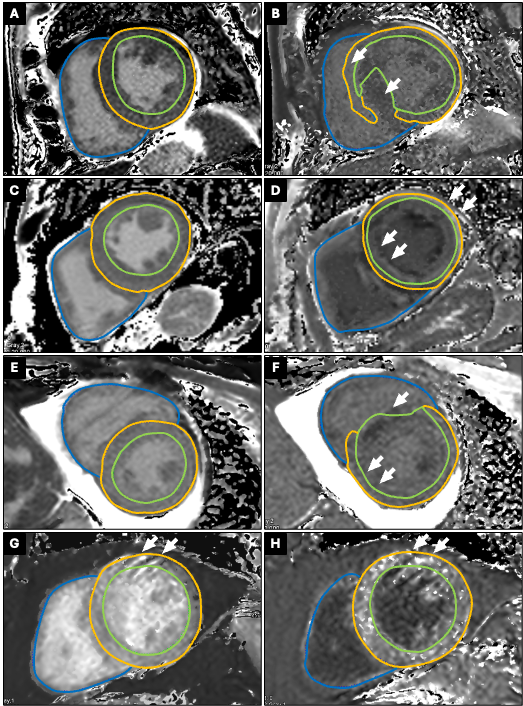


CMR imaging of pre T1 maps (A+C+E+G) and post T1 maps (B+D+F+H) showing various quality issues and findings. (A+B) Significant motion artifacts are observed in the post-contrast T1 mapping, suggesting uncorrected movement. These issues could be addressed during raw data processing for better accuracy. (C+D) Evidence of amyloidosis is seen, characterized by poor contrast between the myocardium and blood, which complicates segmentation of myocardial tissue. (E+F) Further signs suggestive of amyloidosis, with similar contrast-related issues observed. (G+H) Anterior foldover artifact is present, although the myocardial contours remain relatively intact and are considered acceptable despite of the artifact.

# Full-slice to Full-slice analysis of the validation cohort

In these following additional analyses the 325 validation cohort cases were contoured manually using a full-slice delineation in order to find the true performance of the automated contouring and the true difference in ECV values due to segmentation.

## Descriptive contouring assessment using DICE score and Hausdorff distance

| **Variable** | **N** | **Overall**  **n = 325^1^** | **1.5T**  **n = 132^1^** | **3T**  **n = 193^1^** |
| --- | --- | --- | --- | --- |
| **DICE similarity coefficient of Myocardials Area** | 325 | 0.93 ± 0.05 (0.92, 0.94) | 0.94 ± 0.04 (0.93, 0.94) | 0.92 ± 0.06 (0.92, 0.93) |
| **DICE Dice similarity coefficient of Blood Pool Area** | 325 | 0.991 ± 0.013 (0.99, 0.99) | 0.991 ± 0.012 (0.99, 0.99) | 0.991 ± 0.014 (0.99, 0.99) |
| **Hausdorff distance of Epicardial Contours** | 325 | 1.90 ± 0.98 (1.8, 2.0) | 1.72 ± 0.99 (1.5, 1.9) | 2.02 ± 0.95 (1.9, 2.2) |
| **Hausdorff distance of Endocardial Contours** | 325 | 0.96 ± 1.06 (0.85, 1.1) | 0.85 ± 0.83 (0.71, 1.0) | 1.04 ± 1.19 (0.87, 1.2) |
| ^1^Mean ± SD (95% Confidence Interval) | | | | |

### Dice similarity coefficient (DICE Score) - Boxplot

### Hausdorff distance - Boxplot

## Full-slice vs. full-slice ECV - means ± SD and paired t-test

*ECV value based on segmentation approach and hematocrit sampling method.*

| **Field Strength** | **Label** | **Contouring Method** | **Hematocrit Method** | **ECV Value*^a^*** | **p-value *^b^*** |
| --- | --- | --- | --- | --- | --- |
| **1.5T** | Full-slice Manual Synthetic ECV | Manual Full Slice | Synthetic | 28.6 ± 5.3 | **0.003** |
|  | Fully Automated Synthetic ECV | Automated | Synthetic | 28.3 ± 4.8 |  |
| **3T** | Full-slice Manual Synthetic ECV | Manual Full Slice | Synthetic | 26.1 ± 4.9 | 0.067 |
|  | Fully Automated Synthetic ECV | Automated | Synthetic | 26.0 ± 4.7 |  |
| **All** | Full-slice Manual Synthetic ECV | Manual Full Slice | Synthetic | 27.1 ± 5.2 | **<0.001** |
|  | Fully Automated Synthetic ECV | Automated | Synthetic | 26.9 ± 4.9 |  |
| *^a^* reported in mean ± SD  *^b^* paired t-test | | | | | |

### Boxplot with paired t-test

## Bland Altman Analysis of Full-slice manual synthetic ECV vs. fully automated synthetic ECV

### Bland Altman Analysis of Full-slice manual synthetic ECV vs. fully automated synthetic ECV for the full validation cohort

### Bland Altman Analysis of Full-slice manual synthetic ECV vs. fully automated synthetic ECV < 35%

### Bland Altman Analysis of Full-slice manual synthetic ECV vs. fully automated synthetic ECV > 35%

## Regression Models

### Regression Model of Full-slice manual synthetic ECV vs. fully automated synthetic ECV

### Regression Model of Full-slice manual synthetic ECV vs. fully automated synthetic ECV per scanner

## Contingency tables for diagnostic agreement


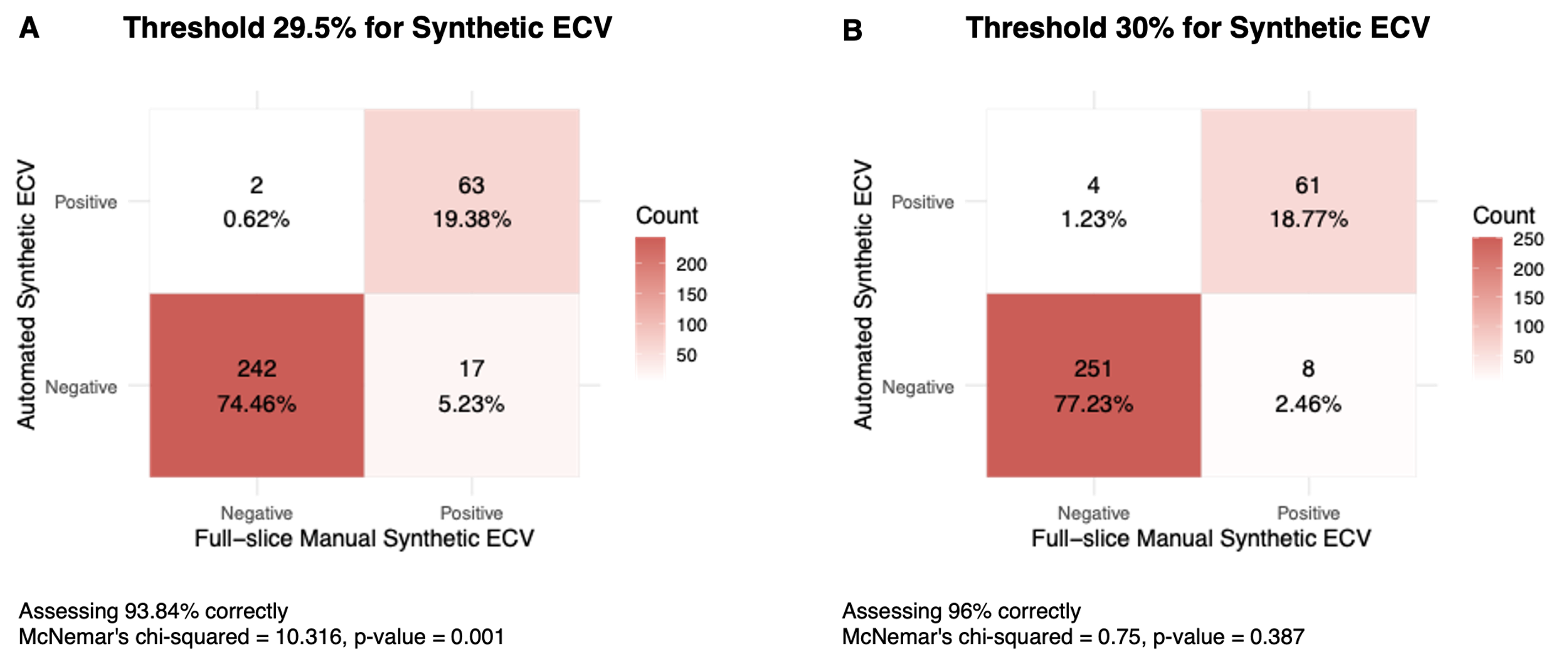


For both tests, a threshold of 30% was used for full-slice manual synthetic ECV and the threshold as indicated for fully automated synthetic ECV (Synthetic ECV)
